# The relationship between physiological biomarkers, physical function, and fall‐risk among people living with dementia

**DOI:** 10.1002/alz.083897

**Published:** 2025-01-09

**Authors:** Sophia N Hamill, Yanbin Dong, Haidong Zhu, Ying Huang, Andre Soares, Jennifer L Waller, Lufei Young, Dawnchelle Robinson‐Johnson, Richard Sams, Mark Hamrick, Deborah A Jehu

**Affiliations:** ^1^ Augusta University, Augusta, GA USA; ^2^ University of North Carolina, Charlotte, NC USA; ^3^ Claiborne Assisted Living Facility, Augusta, GA USA; ^4^ Georgia War Veterans Nursing Home, Augusta, GA USA

## Abstract

**Background:**

People living with dementia (PWD) have upregulated inflammatory pathways, exaggerated metabolic aging, and cellular aging. They also have declines in physical function and heightened fall‐risk. Understanding the physiologic factors that influence physical decline and fall‐risk in PWD is vital to assess and prevent adverse health outcomes, such as future falls. The purpose of this study was to explore the association between physiological biomarkers, physical decline, and fall‐risk in PWD.

**Method:**

In this cross‐sectional study, we used the baseline data of n=42 PWD in residential care facilities from our pilot randomized controlled trial [NCT05488951]. We assessed fall‐risk with the Morse Fall Scale and pulled fall history in the last 6 months from incident reports in medical charts. Participants completed two 4‐meter usual pace walking trials. We assessed two trials of maximum quadriceps strength on each leg with a portable dynamometer. We drew fasted blood and measured inflammatory biomarkers (Interleukin(IL)‐1b, IL‐6, IL‐8, IL‐10, IL‐12p70, IL‐17A, IL‐18, IL23, IL‐33, chemokine ligand 2, tumor necrosis factor‐a, human interferon (INF)‐a2, INFg), metabolic aging (kynurenine), and cellular aging (telomere length). Separate multiple linear regressions were performed for each biomarker, with gait speed, leg strength, fall history, and the Morse Fall Scale as variables of interest. We controlled for age, sex, and the Montreal Cognitive Assessment in each model.

**Result:**

Fall history (β=5.61, p=0.03) and older age (β=0.49, p=0.005) were associated with greater INF‐a2 (R^2^=0.49, p=0.07). Fall history (β=4.93, p=0.07) showed a trend for a relationship with greater IL‐10 (R^2^=0.50, p=0.04). Older age (β=0.28, p=0.009) and lower MOCA scores (β=‐0.40, p=0.04) were related to greater IL‐12p70 (R^2^=0.64, p=0.003). Older age (β=9.26, p=0.01), fall history (β=74.42, p=0.04), and poorer leg strength (β=‐7.40, p=0.06) were related to greater kynurenine (R^2^=0.49, p=0.02).

**Conclusion:**

Our exploratory findings suggest that there may be a relationship between certain physiological biomarkers (INF‐a2, IL‐10, kynurenine), physical function, and fall history. These inflammatory and metabolic aging biomarkers may play an important role for physical function and fall‐risk in PWD. This preliminary research may have implications for screening and monitoring of physical decline and fall‐risk among PWD.

